# Age-Related Macular Degeneration and the Aging Brain

**DOI:** 10.1093/geroni/igab046.598

**Published:** 2021-12-17

**Authors:** Heather Whitson, Alexandra Badea, Jacques Stout, Simon Davis, Jie Zhuang, David Madden

**Affiliations:** 1 Duke University School of Medicine, Durham, North Carolina, United States; 2 DUKE UNIVERSITY, Durham, North Carolina, United States; 3 Shanghai University of Sport, Durham, North Carolina, United States

## Abstract

Age-related macular degeneration (AMD), a leading cause of vision loss in older Americans, is associated with cognitive decline and, particularly, worse performance on verbal fluency tasks. To determine whether AMD is associated with changes in brain structure that may underlie decline in cognition, we conducted a longitudinal, observational study of 39 visually impaired AMD patients and 33 age-matched peers with healthy eyes. Participants (mean age 74.3) underwent cognitive assessments and 3T magnetic resonance imaging (MRI) at baseline and two years. At baseline, AMD patients exhibited lower cortical volume and worse white matter tract integrity, especially in inter-hemispheric connections (FDR <0.05). Principal components analyses revealed faster white matter decline in the AMD group, especially in visual cortex and left hemisphere, which is implicated in language tasks. Understanding patterns of regional brain atrophy in AMD sheds light on mechanisms for the AMD-cognition link and opens windows of opportunity for intervention.

